# Genome Wide Identification and Characterization of LIM Domain Associated Gene Superfamily in Common Bean (*Phaseolus vulgaris* L.)

**DOI:** 10.1155/ijog/5584901

**Published:** 2025-12-10

**Authors:** Raiyyan Abdur Rahim, Maria Chowdhory, Mahmuda Akhter Hasi, Fabiha Haque, Arif Hasan Khan Robin

**Affiliations:** ^1^ Department of Genetics and Plant Breeding, Bangladesh Agricultural University, Mymensingh, Bangladesh, bau.edu.bd; ^2^ Bangladesh Rice Research Institute (BRRI), Gazipur, Bangladesh, brri.gov.bd

**Keywords:** common bean, LIM, phylogenetic analysis, protein interaction networks, sequence comparison, stress response

## Abstract

*LIM genes* are essential for cytoskeletal organization and cellular stress responses in plants. This study conducted a comprehensive genome‐wide investigation of LIM domain‐associated genes in *Phaseolus vulgaris*. This study expands LIM gene classification beyond traditional domains to include LIM_bind factors, revealing that *P. vulgaris* has a more complex and functionally diverse regulatory toolkit than previously understood. Seventeen identified *PvLIM genes* are distributed across three families: LIM‐LIM domain, LIM‐DA1_like domain, and LIM_bind domain‐containing. Following gene duplication, *P. vulgaris LIM genes* underwent subfunctionalization where ancestral functions were partitioned among copies, creating specialized tissue‐specific and stress‐responsive roles while maintaining complementary regulatory networks. PvLIM proteins form critical networks controlling cell wall metabolism, signaling pathways, actin cytoskeleton dynamics, and endocytosis. All *PvLIM genes* exhibit universal responsiveness to multiple stress stimuli including abscisic acid, ethylene, methyl jasmonate, wound, heat, and anaerobic stress conditions, confirming their central role in plant stress tolerance mechanisms. Gene expression analysis revealed tissue‐specific functional specialization, with LIM‐LIM and LIM_bind domain genes predominantly active in stem and leaf tissues, while PvLIM14 governs stem, pod, and leaf development and PvLIM16 modulates seed‐related processes. *PvLIM genes* constitute essential molecular machinery governing plant growth, development, and stress adaptation through integrated phytohormone‐mediated regulatory networks.

## 1. Introduction

Plant stress tolerance mechanisms determine global crop productivity and food security. Transcription factors regulate cellular responses to environmental challenges through complex protein networks. Among these regulatory proteins, LIM domain‐containing transcription factors play essential roles in stress adaptation and developmental processes across plant species [1–4]. These zinc finger motif‐containing proteins facilitate protein‐protein interactions that control gene expression during critical cellular events [5].

The family name comes from three initial homeodomain proteins discovered: LIN11, ISL1, and MEC3 [6–9]. These proteins contain conserved cysteine‐histidine‐rich zinc‐binding regions consisting of two adjacent zinc finger motifs connected by a hydrophobic two‐amino‐acid spacer [5, 10–12]. Particularly in plants, these proteins are found across multiple cellular compartments (cytoplasm, nucleus, nucleoplasm) and manage stress response genes while coordinating metabolic processes, cellular signaling, and structural organization [1–3, 13]. The modular protein‐binding interface follows a consensus sequence [C‐X2‐C‐X16–23‐H‐X2‐C]‐X2 [C‐X2‐C‐X16–21C‐X2–3‐(C/D/H)] and ranges from 50 to 60 amino acids in length [5].

Plant LIM proteins are grouped into two main categories determined by their domain count: single‐domain types (DAR/DA1) and dual‐domain types (2LIMs) 14,15. DAR and DA1 groups possess one unique LIM domain and exist exclusively in plant species [14]. The 2LIM proteins feature two LIM domains with an intervening sequence of roughly 50 amino acids [15]. These proteins contain two ubiquitin‐binding motifs (UIMs), one LIM domain, and a preserved terminal region [14]. This study proposes expanding this classification by defining the LIM domain‐associated gene superfamily to include genes possessing either LIM domains or LIM_bind domains, as LIM_bind domain proteins produce binding proteins that relate closely to overall LIM protein activity [16, 17].

Current research shows that LIM proteins initiate actin generation during plant growth and development [18]. Under abiotic stress conditions, significant amounts of LIM proteins bind to F‐actin for cytoskeletal structure allocation, maintaining physiological processes through mechanosensing [19–21].

The discussion of *LIM genes* in plants began with the discovery of HaP‐LIM1 in sunflower pollen [15]. In *Arabidopsis thaliana* and *Oryza sativa*, six CRP‐like LIM proteins associate with actin filament formation to prevent cytoskeletal breakdown [18, 22]. LIM proteins support lignin production in tobacco plants 25 and promote fiber cell growth plus secondary wall formation in cotton [23].

Common bean (*Phaseolus vulgaris* L.) represents an important legume crop enriched in protein, carbohydrates, and essential nutrients [24, 25]. Adverse weather patterns, however, cause substantial decreases in common bean production [26]. Global bean production faces threats from combined biotic–abiotic stresses, placing 400 million people who depend on beans for iron and protein at risk [27]. Biotic stress alone causes 30%–61% yield reduction, resulting in substantial economic losses [28]. Disease management costs reduce profitability by 15%–30% [27]. Although traditionally linked to plant growth and development, cytoskeletal structures also play a critical role in stress regulation by modulating morphogenesis, signal transduction, cell division, cellular motility, and defense responses.

This study aimed to identify LIM domain‐associated proteins in common bean and explore their involvement in different biological processes. Genetic resilience exploration in *P. vulgaris* represents an urgent requirement given current agricultural challenges. The research focused on comprehensive identification and characterization of *LIM genes* and their proteins within the common bean genome.

Seventeen *LIM genes* were identified and characterized in the common bean genome through thorough *in silico* analysis using different bioinformatics approaches including physicochemical property assessment, phylogenetic analysis, multiple sequence comparison, protein’s structural and interaction analysis, cis element analysis, and expression pattern analysis. This research provides the first comprehensive analysis of LIM domain‐associated proteins in common bean, expanding understanding of stress tolerance mechanisms in legume crops. The findings establish a theoretical foundation for future research on LIM domain‐associated protein functions in legume plants, potentially contributing to improved stress resistance and crop productivity.

## 2. Materials and Methods

### 2.1. Identification of LIM Domain Associated Genes in *P. vulgaris* and Data Retrieval

The latest *P. vulgaris* genome assembly (PhaVulg1_0), coding sequence, GFF annotation information, and protein sequence files were downloaded from Ensembl Plants (https://plants.ensembl.org/) [29] (Supplementary Dataset 1). In the pursuit of a comprehensive identification of all LIM domain associated genes within *P. vulgaris*, the *A. thaliana* LIM gene domain associated protein sequences and annotation information were downloaded from the TAIR (http://www.arabidopsis.org/) database. The *P. vulgaris LIM genes* were predicted in the genome using alignment based BLASTP and BLASTN search and a hidden Markov model‐based query (HMMER) with known sequences of *A. thaliana* and *Glycine max LIM genes* using TBTools [30] and the HMMER web server (https://www.ebi.ac.uk/Tools/hmmer/), respectively. The pfam and interpro IDs obtained from the HMMER search were subsequently used to retrieve specific genes. On the other hand, more than 300 unique hits were found after a BLASTp was performed (e < 1e‐5) on the *P. vulgaris* genome against all known AtLIM proteins.

For the purpose of a more accurate identification of *PvLIM genes*, the presence of the LIM domain or LIM_bind domain were predicted in 17 *PvLIM genes* using motif search in PROSITE format and the Pfam database using the Genome JP web server (https://www.genome.jp/). Additionally, an NCBI CD (NCBI Conserved Domain Search (nih.gov)) search was also performed to confirm the presence of LIM domains. The Genome JP web motif search, NCBI CD search, and search in the UniProt database confirmed 17 *PvLIM genes* to be part of the LIM domain associated gene superfamily (e value < 1e^−5^). Only one protein and coding sequence were selected for analysis from genes having more than one transcript, on the basis of the Ensembl Cannonical preference. With the help of the UniProt database (https://www.uniprot.org/), the presence of the domains was crosschecked by manual search. The genes were then assigned with Ensembl Plant gene IDs and categorized based on their domain composition. A basic gene model was prepared on the basis of patterns of the categorized domains.

Additionally, the protein sequences of *A. thaliana* (18), *Brassica rapa* (24), *Chenopodium quinoa* (14), *G. max* (42), *Vigna radiata* (18), and *Solanum lycopersicum* (18) were collected using Ensembl Genome Database [29], from the TAIR10 assembly, SCU_BraROA_2.3 assembly, ASM168347v1 assembly, Glycine_max_v2.1 assembly, Vradiata_ver6 v, and SL3.0 assembly, respectively.

### 2.2. Physicochemical Properties Analysis and Subcellular Localization of PvLIM Proteins

The ExPASy ProtParam [31] online tool was utilized to assess the fundamental physicochemical properties of the PvLIM proteins using default settings. Parameters that were analyzed included amino acid count, molecular weight, theoretical isoelectric point (pI), instability index, aliphatic index, and the grand average of hydropathicity (GRAVY).

To predict the subcellular localization of PvLIM proteins, the CELLO predictive model (C. [32]) (http://cello.life.nctu.edu.tw/) and Cell‐PLoc [33] (http://www.csbio.sjtu.edu.cn/bioinf/CellPLoc-2/) web tools were employed. In the case of data predicted by CELLO, only the significant hit predictions (having Asterix) were considered.

### 2.3. Chromosome Location and Gene Duplication Analysis

The distribution of *PvLIM genes* on the 11 *P. vulgaris* chromosomes was analyzed from the annotation information of the *PvLIM genes*. The analysis of duplication was performed by analyzing BLASTn results of the *PvLIM genes* using TBtools [30]. This study employed a combination of methodologies drawn from various previous research efforts to detect gene duplication [34]. Gene pairs with > 80% sequence identity and > 60% query coverage was classified as duplicates. Secondary selection was based on exon‐intron similarity, and final duplication confirmation relied on synteny analysis with > 75% sequence similarity. In the case of duplication, if the positions of the genes were on the same chromosome and positioned adjacently, they were considered tandem duplication. Proximal duplications were defined as non‐tandem genes on the same chromosome with no more than 20 annotated genes between each other. Genes positioned on different chromosomes were classified as segmental duplication. Ka/Ks value of the paralogs were calculated to predict if the mutation was purifying (if ka/ks > 1), neutral (if ka/ks = 1) or diversifying (if ka/ks < 1).

### 2.4. Gene Structure and Conservative Motif Analysis

The GSDS 2.0 web tool [35] was utilized in this study to analyze and visualize exon–intron distribution of *PvLIM genes* from gDNA and CDS data. The gDNA data was extracted from the downloaded *P. vulgaris* genome assembly (PhaVulg1_0) using TBTools [30]. The members with higher homology were placed closer to visualize the structural similarities among those.

The MEME suite v5.5.6 was employed to analyze the presence of protein motifs in identified *PvLIM genes* [36].The analysis parameters were configured with a limit of 10 motifs, where a minimum motif length of six, and a maximum length of 50 were employed.

### 2.5. Evolutionary Analysis and Synteny Analysis of PvLIM Proteins

A phylogenetic tree was constructed using the Neighbor‐Joining (NJ) method in MEGA11, which integrated inbuilt protein sequence alignment by default [37] and applying the Bootstrap method with 1000 replicates. After constructing the tree, the *PvLIM genes* were classified based on their phylogenetic relationships.

To identify syntenic regions across *P. vulgaris*, *A. thaliana*, *B. rapa*, *C. quinoa*, *G. max*, *V. radiata*, and *S. lycopersicum*, gene sequences were uploaded to the Circoletto tool (https://bat.infspire.org/circoletto/). Syntenic regions were identified using default Circoletto parameters, with ribbon coloration based on E‐values, and a microsynteny map was created using the same web‐based platform [38]. This study excluded the weak links in the synteny map that had less than 75% similarities.

### 2.6. Multiple Sequence Alignment, Protein Homology Modeling, and Construction of Protein Interaction Network Diagram

Protein sequences of *P. vulgaris* (PvLIM), *A. thaliana* (AtLIM), *B. rapa* (BrLIM), *C. quinoa* (CqLIM), *G. max* (GmLIM), *V. radiata* (VrLIM), and *S. lycopersicum* (SlLIM) were aligned using ClustalX v2 [39]. The resulting alignment file was then exported to Genedoc (Nicholas & [40]) for visualization and illustration of the sequence alignment.

The three‐dimensional structures of LIM proteins were modeled using SWISS‐MODEL (https://www.swissmodel.expasy.org) [41] to predict their tertiary structural homology.

Additionally, the protein–protein interaction networks of PvLIM proteins were predicted through the STRING database (https://string-db.org/) [42].

### 2.7. Analysis of Promoter Cis‐Acting Elements and Functional Prediction

The PlantCARE (http://bioinformatics.psb.ugent.be/webtools/plantcare/html/) database [43] was utilized to identify and categorize the *cis-*acting promoter elements, typically 5 to 10 base pairs in length, of the *PvLIM genes*. The resulting data were visualized as a heat map using TBtools.

Gene ontology (GO) predictions were inferred from electronic annotations available in the Ensembl Plants database. The specific functional roles of the genes were further explored and analyzed using the AmiGO 2 web tool (https://amigo.geneontology.org/amigo), [44, 45].

### 2.8. Expression Data Analysis

Expression data of *PvLIMs* were taken in the format of median normalized reads per kilobase per million mapped reads (RPKM) values from the *P. vulgaris* Gene Expression Atlas under accession number SRP046307 (https://www.zhaolab.org/PvGEA/) [46].

PhaVulg1_0 gene IDs were converted to Pvulgaris_442_v2.1 database identifiers to utilize PvGEA resources. Initially, the DAVID tool converted ensemble IDs to UniProt accessions, which were then queried in PhytoMine, resulting in 11 matches from the original 17 target genes. To ensure complete coverage of all 17 genes, protein sequences were retrieved from Phytozome in FASTA format and subjected to BLAST analysis using known bean LIM proteins as reference sequences. Gene IDs were selected through hierarchical criteria: similarity percentage served as the primary factor, followed by E‐value (lower values preferred) when tied, then bit score (higher values preferred) for remaining ties, and finally the first transcript when all parameters were equivalent. This systematic approach ensured reliable protein identification across databases. The analysis revealed that 16 out of 17 genes displayed perfect 100% identity matches, with PvLIM10 showing 96.33% identity as the sole exception, which remained the highest available match. Throughout this process, all genes maintained e‐values consistently below 1e‐140, confirming statistical significance.

A total of 24 types of tissues of *P. vulgaris,* including young leaf, leaf 21 days after emergence of unifoliate (DAE), stem, shoot, flower, young pod, pod 9 cm, pod 10 cm, pod 12 cm, seed 7 mg, seed 50 mg, seed 140 mg, root tip, young root, and root 21 DAE were analyzed. A heat map was generated with these median normalized RPKM values using the heat map function in TBTools [30].

A co‐expression heat map was generated to examine the overall expression correlation among *PvLIM genes*, utilizing Pearson’s correlation coefficient (r) calculated from RPKM values across all aforementioned tissues of *P. vulgaris*.

## 3. Results

### 3.1. Identification of LIM Domain Associated Genes and Data Retrieval

A total of 17 unique *PvLIM genes* were identified in this study using in silico analysis. The domains that were found in *PvLIM genes* were LIM, LIM_DA1, and LIM_bind (Supplementary Table S7). HMMER search using the LIM pfam domain (PF00412 or IPR001781) and LIM_bind pfam family (PF01803 or IPR029005) revealed 13 and four genes, respectively (Supplementary Table S7). This validates the identification of total of 17 found genes.

Specifically, *PvLIM*2, *PvLIM*5, *PvLIM*7, *PvLIM11*, *PvLIM12*, *PvLIM14*, *PvLIM16*, and *PvLIM17* were found to possess two LIM domains, while genes *PvLIM*1, *PvLIM*4, *PvLIM*6, *PvLIM*8, and *PvLIM13* contained a single LIM and DA1_like domain. Additionally, *PvLIM*3, *PvLIM*9, *PvLIM10*, and *PvLIM15* exhibited the presence of a LIM_bind domain. This results in a classification in three groups where Group I, Group II, and Group III consist of genes having double LIM domains, one LIM and one DA1_like domain, and one LIM_bind domain, respectively (Supplementary Figure S1, Supplementary Table S7).

### 3.2. Basic Physicochemical Properties Analysis and Subcellular Localization of LIM Proteins

The *PvLIM* gene sequences are mapped across *P. vulgaris* chromosomes, specifically on the chromosome number one, two, three, four, seven, eight, nine, and ten. The nucleotide position of *PvLIM genes*, where the protein sequences begin and where those end, indicated their position within the chromosome (Table 1). The proteins vary in size, ranging from 187 amino acids (PvLIM11) to 913 amino acids (PvLIM15). The smallest protein is PvLIM11, while PvLIM15 is the largest (Table 1). The molecular weight of these proteins ranges from as light as 21.13 kDa (PvLIM5) to as heavy as 99.66 kDa (PvLIM15) (Table 1). The average molecular weight of Group I, Group II, and Group III were 22.45 kDa (kilodaltons), 59.26 kDa, 97.77 kDa, respectively, making group the heaviest and largest proteins (Table 1). The theoretical isoelectric pH values, representing the isoelectric points of the proteins, vary from as low as 5.17 (PvLIM8) to as high as 9.21 (PvLIM7) (Table 1). The average isoelectric pH values for the groups were as follows: Group I had a pH of 8.21, Group II had a pH of 6.46, and Group III had a pH of 9.03 (Table 1). Proteins with higher values are more basic in nature. The level of stability of a protein in in vitro condition is predicted by the protein instability index [47]. Among the 17 proteins, six were found as stable, while the rest were unstable (Table 1). The relative volume of aliphatic side chains, also known as aliphatic index is considered a factor contributing to protein thermostability [48]. The aliphatic index values ranged from 48.32 (PvLIM12) to 76.18 (PvLIM10), with higher numbers indicating higher stability in extreme conditions (Table 1). Negative GRAVY values indicate that the protein is hydrophilic, and all proteins of the PvLIM superfamily have negative values, making them hydrophilic (Table 1).

**Table 1:** Properties of PvLIM genes and their corresponding proteins:

**Group**	**Gene ID**	**Gene name**	**Location**				**Protein length**	**Molecular weight (kDA)**∗	**Average molecular weight**	**Isoelectric pH**	**Average isoelectric pH**	**Exon**	**Intron**	**Instability index**	**Stability class**	**Aliphatic index**	**GRAVY**∗	**Subcellular location**	
			**Chr. No** ^a^	**Start**	**End**	**Length**			**(kDA)**∗									**CellPLoc**	**Cello**
One	PHAVU_001G239700g	*PvLIM*5	1	49987707	49990679	2972	194	21.13	22.45	9.08	8.21	5	4	38.4	Stable	52.47	‐0.443	Golgi apparatus, Nucleus	Nuclear, Extracellular
	PHAVU_007G046100g	*PvLIM11*	7	3683207	3685582	2375	187	21.22		8.94		5	4	31.85	Stable	57.27	‐0.598	Golgi apparatus, Nucleus	Extracellular
	PHAVU_009G181200g	*PvLIM16*	9	26696676	26700497	3821	191	21.65		8.83		5	4	24.52	Stable	56.65	‐0.625	Golgi apparatus, Nucleus	Extracellular
	PHAVU_001G028200g	*PvLIM*2	1	2617498	2619665	2167	196	21.74		8.81		5	4	25.7	Stable	56.73	‐0.598	Golgi apparatus, Nucleus	Nuclear, Cytoplasmic, Extracellular
	PHAVU_009G072200g	*PvLIM14*	9	12021375	12023626	2251	196	21.76		8.93		5	4	20.03	Stable	58.72	‐0.587	Golgi apparatus, Nucleus	Extracellular, Cytoplasmic
	PHAVU_003G197600g	*PvLIM*7	3	41030989	41034056	3067	204	23.19		9.21		5	4	29.41	Stable	60.64	‐0.586	Nuclear	Nuclear, Extracellular
	PHAVU_008G067500g	*PvLIM12*	8	6112188	6114287	2099	208	23.56		6.26		5	4	52.65	Unstable	48.32	‐0.717	Nuclear	Nuclear, Extracellular
	PHAVU_010G095700g	*PvLIM17*	10	34846438	34848179	1741	226	25.41		5.6		5	4	48.52	Unstable	53.5	‐0.628	Nuclear	Nuclear
Two	PHAVU_001G017100g	*PvLIM*1	1	1429859	1434928	5069	469	53.65	59.26	6.14	6.46	11	10	50.69	Unstable	65.99	‐0.624	Nuclear	Nuclear
	PHAVU_001G237100g	*PvLIM*4	1	49738889	49744752	5863	511	57.98		8.22		12	11	52.86	Unstable	66.09	‐0.557	Nuclear	Nuclear
	PHAVU_003G250800g	*PvLIM*8	3	47788365	47794718	6353	529	60.06		5.17		11	10	57.45	Unstable	68.05	‐0.685	Nuclear	Nuclear
	PHAVU_008G229800g	*PvLIM13*	8	54461684	54466341	4657	539	60.93		7.55		12	11	55.1	Unstable	70.07	‐0.435	Nuclear	Plasma Membrane, Nuclear
	PHAVU_002G024900g	*PvLIM*6	2	2660954	2668059	7105	558	63.7		5.23		11	10	57.05	Unstable	70.65	‐0.706	Nuclear	Nuclear
Three	PHAVU_004G121300g	*PvLIM*9	4	39098917	39105192	6275	859	93.88	97.77	9.02	9.03	9	8	64.06	Unstable	73.91	‐0.551	Nuclear	Nuclear
	PHAVU_004G121400g	*PvLIM10*	4	39114511	39119247	4736	900	98.5		9.08		8	7	61.6	Unstable	76.18	‐0.495	Nuclear	Nuclear
	PHAVU_001G064200g	*PvLIM*3	1	8009739	8021477	11738	910	99.05		8.97		9	8	61.06	Unstable	63.56	‐0.688	Nuclear	Nuclear
	PHAVU_009G134000g	*PvLIM15*	9	19713596	19722986	9390	913	99.66		9.05		9	8	59.56	Unstable	64.6	‐0.644	Nuclear	Nuclear

^a^Chr no – Chromosome number ∗‐ kDA‐ kilodaltons, ∗GRAVY‐Grand Average of Hydropathy.

The prediction of subcellular localization of PvLIMs was mostly in the nucleus of the cell except for two discrepant results (Table 1). There was no significant prediction of nuclear localization for PvLIM11 and PvLIM14 in the CELLO predictive system. However, these two proteins were found to have nuclear localization as their second most significant prediction in CellPLoc prediction (Table 1).

### 3.3. Chromosome Location and Gene Duplication Analysis

**Figure 1:**
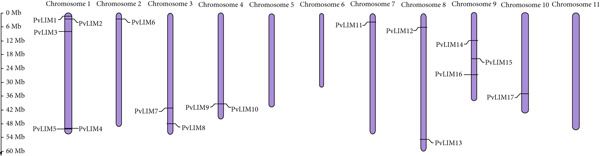
Position of PvLIM genes on chromosomes of *Phaseolus vulgaris*.

The 17 *PvLIM genes* are located on eight out of 11 *P. vulgaris* chromosomes. Chromosome 1 hosts the highest number of *PvLIM genes*, including *PvLIM*1, *PvLIM*2, *PvLIM*3, *PvLIM*4, and *PvLIM*5. Chromosome 2 contains only *PvLIM*6, while Chromosome 3 is home to *PvLIM*7 and *PvLIM*8. Chromosome 4 carries *PvLIM*9 and *PvLIM10*, and Chromosome 7 contains *PvLIM11*. Chromosome 8 has two genes, *PvLIM12* and *PvLIM13*, while Chromosome 9 holds *PvLIM14*, *PvLIM15*, and *PvLIM16*. The last gene, *PvLIM17*, is hosted by chromosome 10 (Figure 1). This distribution shows that some chromosomes, particularly Chromosome 1, have multiple *PvLIM genes* (4), while Chromosomes 5, 6, and 11 host none of the *PvLIM genes* (Figure 1).

Initially, 14 pairs of *PvLIM genes* were predicted to be duplicated based on similarity coverage and percent identity (Supplementary Table S1a). However, after further analyzing gene structure and syntenic relationship, the final selection confirmed a total of five duplicated gene pairs (Supplementary Table S1b). Four of them were segmental duplications, and one was a tandem duplication (Supplementary Table S1b). Their Ka/Ks values vary from 0.09 to 0.28 (Supplementary Table S1b), which are all less than one, indicating that they were subjected to purification selection during the evolution process. Data generated on identity, coverage, exon–intron similarity, and synteny‐based similarity are presented in Supplementary Dataset 2.

### 3.4. Gene Structure and Conservative Motif Analysis

**Figure 2:**
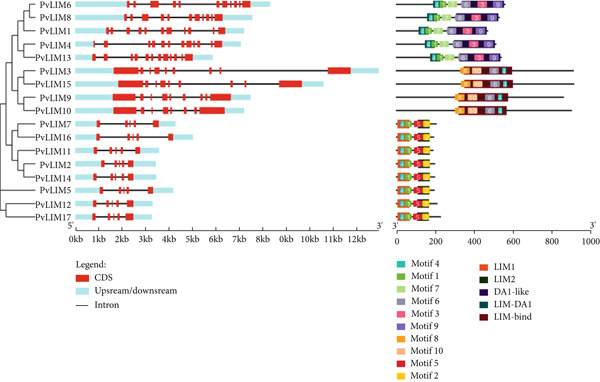
Exon–intron distribution of 17 PvLIM genes predicted from GSDS 2.0 (Left). Ten identified Motif distribution over 17 PvLIM genes alongside their respective domains using Meme Suite (Right).

The gene structural analysis revealed that the *PvLIM genes* having sequence similarities also had structural similarity in terms of exon–intron distribution as well as in present motifs and their distribution. In the analysis, all genes contained more than one exon (Table 1) and no monoexonic gene was found (Figure 2). Although *PvLIM13* had the maximum exon number (12), it neither had the longest sequence nor produced the heaviest protein (Table 1), as the exon lengths of *PvLIM13* were smaller than those of *PvLIM*3 or *PvLIM15* (Figure 2).

In the motif analysis, where 10 different motifs were found to be present in the 17 *PvLIM genes*, three patterns of distribution followed (Figure 2). In pattern 1, Motifs 4, 1, 7, 3, and 9 were observed in the mentioned sequence in *PvLIM*6, *PvLIM*8, *PvLIM*1, *PvLIM*4, and *PvLIM13*. In pattern 2, Motifs 8, 10, 6, and 4 were observed in the mentioned sequence in *PvLIM*3, *PvLIM*5, *PvLIM*9, and *PvLIM10*. In pattern 3, Motifs 4, 1, 5, and 2 were found in the mentioned sequence in *PvLIM*7, *PvLIM16*, *PvLIM11*, *PvLIM*2, *PvLIM14*, *PvLIM*5, *PvLIM12*, and *PvLIM17* (Figure 2). Among the 10 different motifs, Motif 4 was present in all 17 *PvLIM genes*. Motif 8 and Motif 10 were present in the least number of sites (Supplementary Table S9).

### 3.5. Evolutionary Analysis and Synteny Analysis of PvLIM Proteins

**Figure 3:**
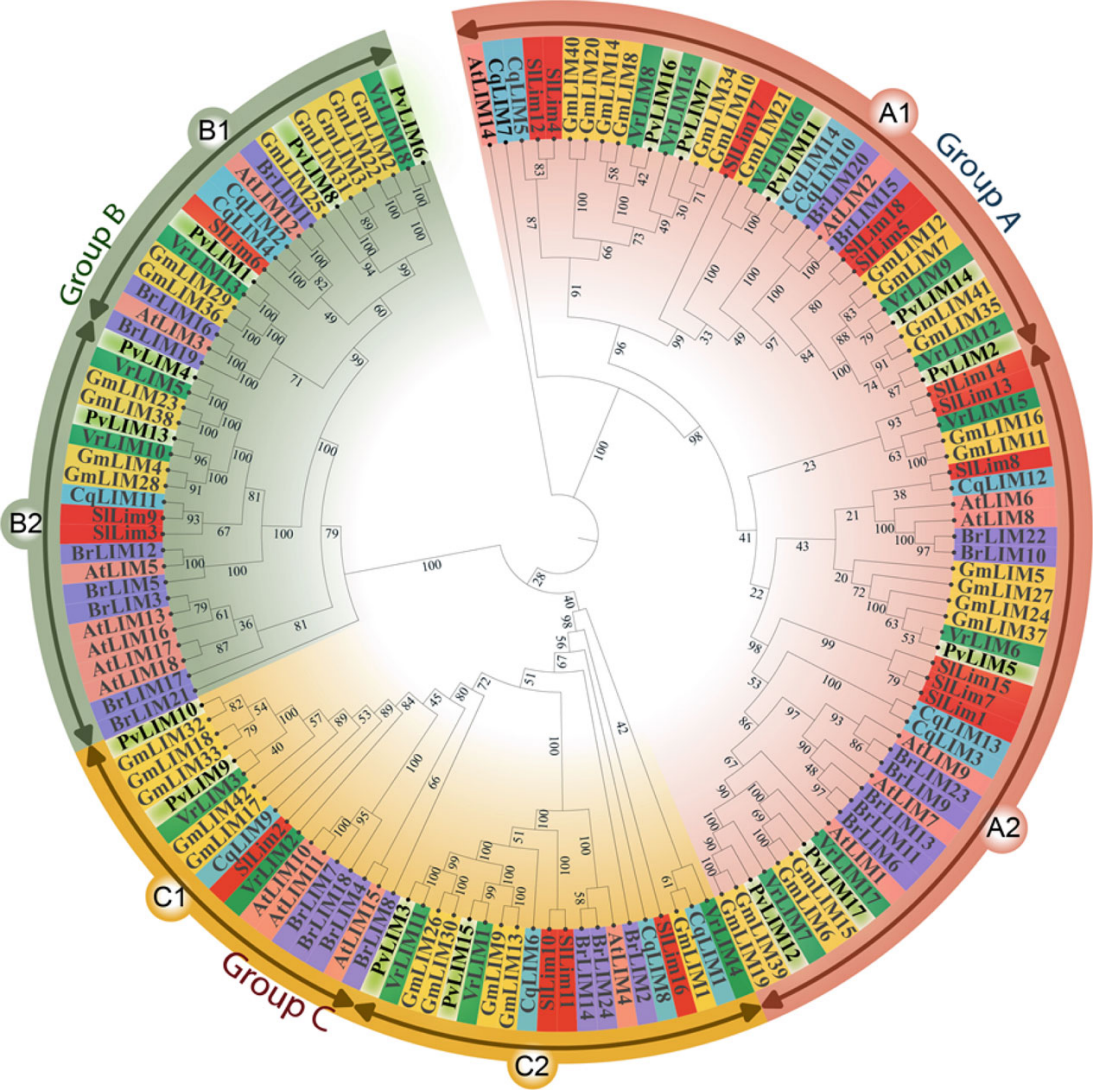
Phylogenetic analysis of LIM proteins from *P. vulgaris*, *A. thaliana*, *B. rapa*, *C. quinoa*, *G. max*, *V. radiata*, and *S. lycopersicum*. The phylogenetic tree was prepared in MEGA‐11 by neighbor joining method with 1000 bootstrap replicates.

For the purpose of a reliable phylogenetic analysis, the generation of a neighbor‐joining tree and visualization of the phylogenetic relationship among the proteins from different species presented three distinct clusters in the relationship tree. These three clusters were then classified into three groups (A–C) (Figure 3). Each group was subsequently divided into two subgroups (A1, A2, B1, B2, C1, C2) based on smaller clusters within the clusters of each group.

Among the proteins from related species, group A contained 72 members, followed by group B (41) and group C (38). All groups contained clusters of LIMs from both monocot and dicot species. The 17 PvLIM proteins were distributed into these three phylogenetic cluster groups. The highest numbers of PvLIM were clustered in group A, containing eight members (Figure 3).

The classification of *PvLIM genes* was performed on the basis of the pattern of present domains in paragraph 4.1 (Supplementary Table S7), which aligned perfectly with the phylogenetic cluster groups (Figure 3). PvLIM proteins from Group I, Group II, and Group III (Supplementary Table S7) were present in the phylogenetic cluster Group A, Group B, and Group C, respectively (Figure 3).

**Figure 4:**
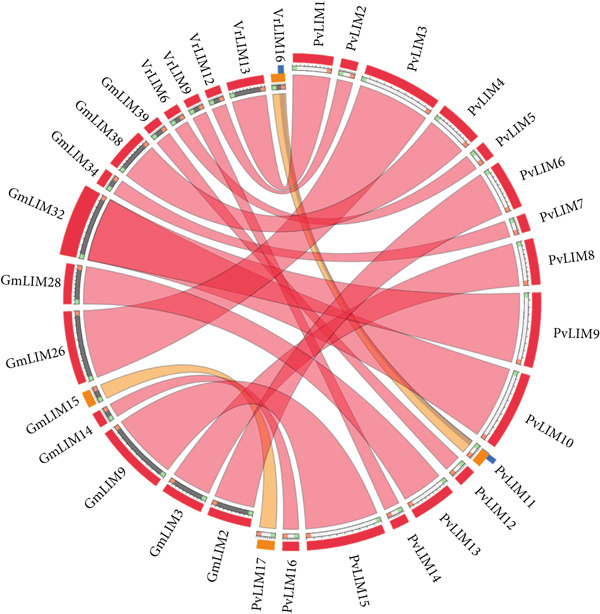
Syntenic relationship among *P. vulgaris*, *A. thaliana*, *B. rapa*, *C. quinoa*, *G. max*, *V. radiata*, and *S. lycopersicum* only showing the best hits using “score/max” ratio coloring. Blue signifies less than 50% similarity (discarded). Green represents 50%–75% similarity (discarded). Orange indicates a similarity range of 75%–99.999% and red denotes 100% similarity.

The syntenic relationship of *PvLIM genes* with other crops revealed that the *PvLIM genes* mostly contain orthologs in *V. radiata* and *G. max* (Figure 4). A total of 11 *GmLIMs* and five *VrLIMs* were found to be orthologs of *PvLIM*s (Figure 4). Both *PvLIM*9 and *PvLIM10* were orthologous to *GmLIM32* (Figure 4). The orthologous pairs of proteins were found at similar positions in the phylogenetic tree as expected (Figure 3).

### 3.6. Multiple Sequence Alignment, Protein Homology Modeling, and Construction of Protein Interaction Network Diagram

Alignment of 17 PvLIM proteins with 134 sequences (Supplementary Dataset 1) from BrLIMs (24), GmLIMs (42), CqLIMs (14), SlLIMs (18), VrLIMs (18), and AtLIMs (18) revealed three conserved patterns across species (Figure 5). Pattern 1 showed two LIM domains with inter‐LIM regions (Group I proteins). Pattern 2 displayed LIM_DA1 and LIM domains. Pattern 3 contained LIM_bind domains (Figure 5).

Figure 5Sequence alignment of LIM proteins in *Phaseolus vulgaris*, *Arabidopsis*, *Brassica rapa*, *Chenopodium quinoa*, *Glycine max*, *Vigna radiata*, and *Solanum lycopersicum*. Regions with 100% similarity is highlighted with black color, dark gray color denotes > 80% similarity and light gray color represents > 60% sequence similarity. (a). Pattern 1 (orange and green colors represent LIM1 and LIM2, respectively and gray color represent inter LIM region), (b) pattern 3 (blue color represent LIM_bind) and (c) pattern 2 (red color represent DAI_like).

**Figure:**
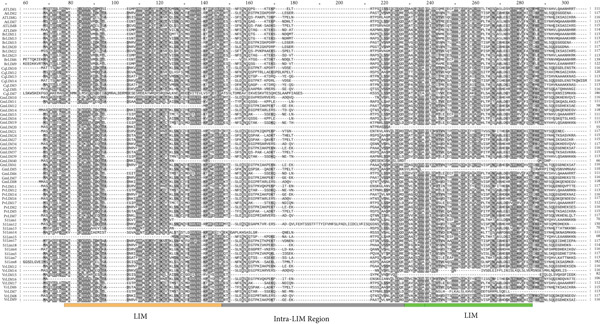
(a)

**Figure:**
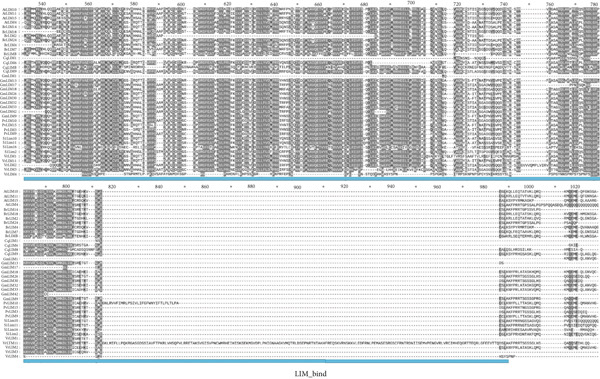
(b)

**Figure:**
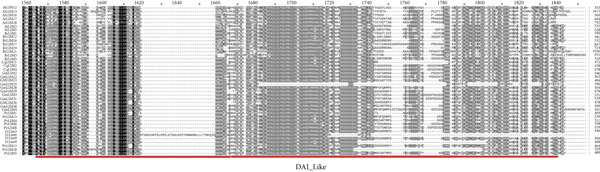
(c)

These patterns strongly correlate with previously identified PvLIM groups from domain analysis (Supplementary Table S7) and phylogenetic clusters (Figure 3). High sequence alignment occurred within phylogenetic groups.

**Figure 6:**
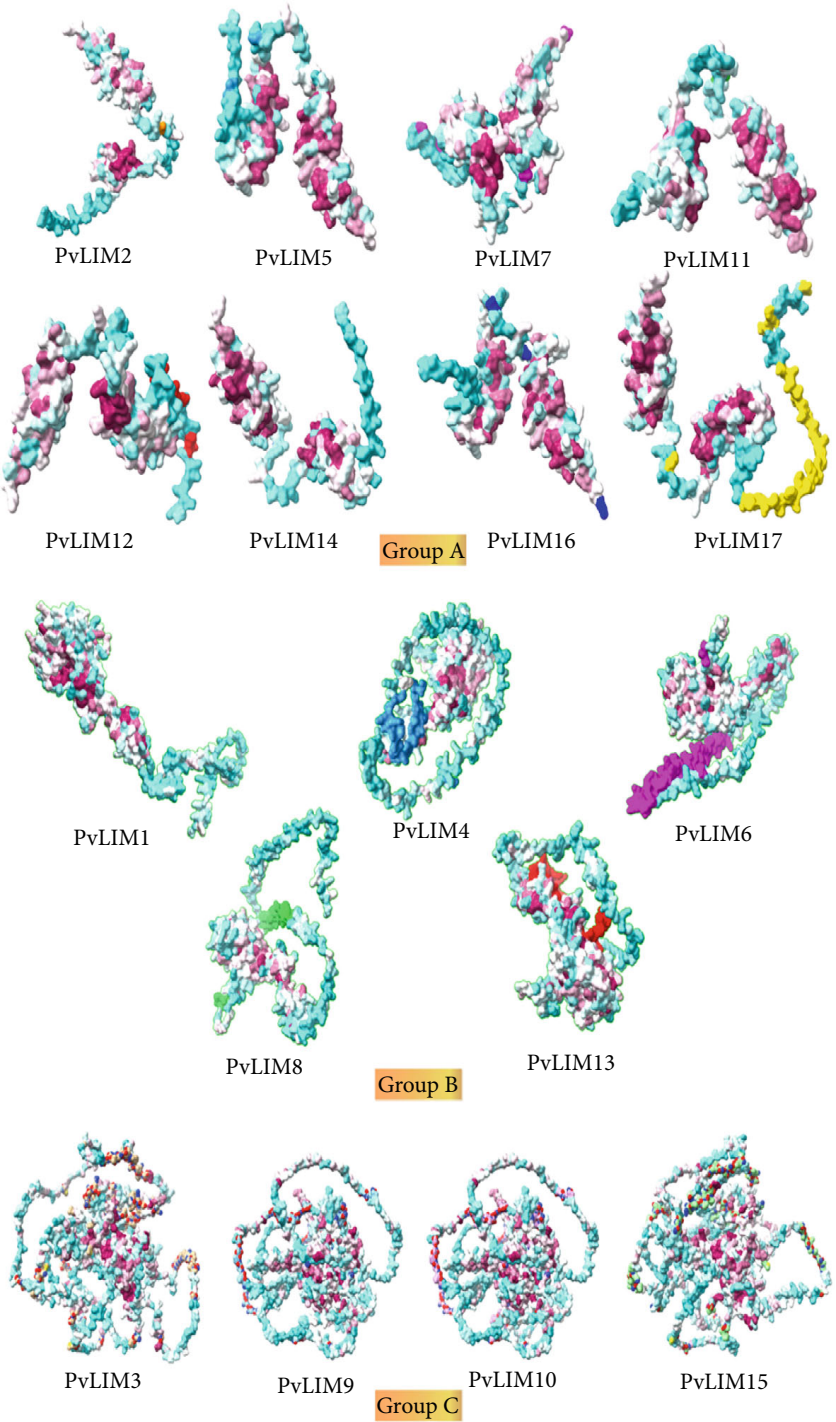
Tertiary structures of all 17 PvLIM proteins. The most highly conserved residues are in maroon and least conserved are in cyan. The log range of the conservation color gradient was from −1.4 to 1.4.

Swiss model prediction of 17 PvLIM proteins showed high structural similarity with characterized groups (Figures 3, 5, Supplementary Table S7, Supplementary Figure S2). Most predicted structures could be superimposed, confirming structural resemblance (Supplementary Figure S2). This tertiary structure similarity suggests functional conservation, though subtle variations indicate potential functional divergence, particularly within group A (Figure 6).

Tertiary modeling showed highest confidence for PvLIM6 and PvLIM8 (Supplementary Table S2), exhibiting significant similarity across analyses (Figure 6). All PvLIM homology models derived exclusively from LIM protein templates of different organisms, predominantly *Glycine* species, suggesting LIM proteins show no detectable tertiary structure similarity with other protein families (Supplementary Table S2).

**Figure 7:**
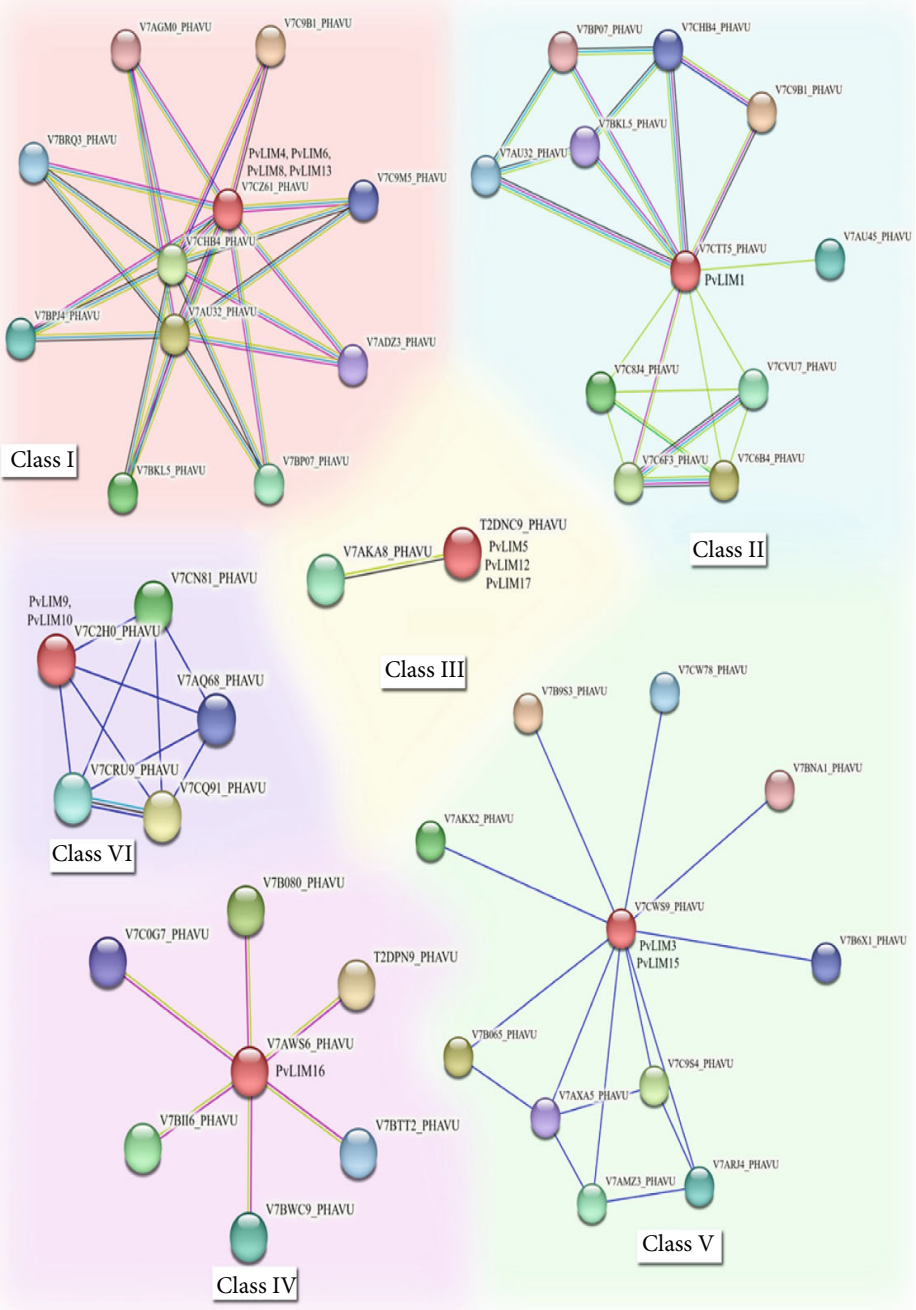
Seven distinguished protein–protein interactions network structure found in *Phaseolus vulgaris* LIM genes. The interacting proteins are marked with their respective uniprot accession. The color scales represent the relative signal intensity scores. Known interactions are distinguished by light blue lines for interactions sourced from curated databases and pink lines for those experimentally determined. Predicted interactions are represented with green lines for gene neighborhood associations, red lines for gene fusions, and dark blue lines for gene co‐occurrence. Additionally, other types of data include text mining results, shown with yellow lines, co‐expression represented by black lines, and protein homology indicated with purple lines.

STRING protein–protein interaction networks revealed *PvLIM* involvement in essential biological functions (Figure 7), classifying PvLIM proteins into seven functional classes.

Class I networks (Figure 7) identified two PF10291 (MHD domain‐containing) and eight PF04885 (STIG1 domain‐containing) proteins. Four InterPro domains were identified: IPR039976 (WPP domain‐interacting tail‐anchored protein), IPR006969 (STIG1), IPR0188080 (Muniscin_C), and IPR028565 (Mu homology domain), linked to male reproductive tissue development (Supplementary Table S3).

Classes I and II shared interactions with MHD domain‐containing proteins, WPP domain‐interacting tail‐anchored protein 2, and stigma‐specific STIG1‐like protein 1. Class II uniquely interacted with Dolichol‐phosphate mannosyltransferase subunit 1, peroxidase, beta‐galactosidase, photolyase/cryptochrome alpha/beta domain‐containing protein, and calcium‐dependent protein kinase, with PvLIM1 as sole representative (Table 2).

**Table 2:** PvLIM genes classification based on protein interaction.

**Class**	**Genes involved**	**Interaction overview**
I	*PvLIM*4, *PvLIM*6, *PvLIM*8, *PvLIM13*	Interacts with MHD (Mu homology domain) containing protein, WPP domain‐interacting tail‐anchored protein, Stigma‐specific protein Stig1, Muniscin C‐terminal containing protein
II	*PvLIM*1	Interacts with MHD domain‐containing protein, Dolichol‐phosphate mannosyltransferase subunit 1, Peroxidase, Beta‐galactosidase, Photolyase/cryptochrome alpha/beta domain‐containing protein, Calcium‐dependent protein kinase, WPP domain‐interacting tail‐anchored protein 2 and Stigma‐specific STIG1‐like protein 1.
III	*PvLIM*5, *PvLIM12*, *PvLIM17*	Interacts with Treslin N‐terminal domain‐containing protein
IV	*PvLIM16*	Interacts with Actin
V	*PvLIM*3, *PvLIM15*	Interacts with SWIM‐type domain‐containing protein, Fip1 domain‐containing protein, Uncharacterized protein, DUF641 domain‐containing protein, Transposase‐associated domain‐containing protein, Trafficking protein particle complex II‐specific subunit 120 homolog,
VI	*PvLIM*9, *PvLIM10*	Interacts with GBF‐interacting protein 1 N‐terminal domain‐containing protein, PORR domain‐containing protein, PAS domain‐containing protein.
VII	*PvLIM*2, *PvLIM*7, *PvLIM11*, *PvLIM14*	No interaction

Class III distinctly interacted with Treslin N‐terminal domain‐containing proteins (Table 2). Class IV exclusively interacted with actin (Table 2). Classes V and VI protein interactions were limited to co‐occurrence only (Figure 7). Class V interacted with SWIM‐type domain‐containing protein, Fip1 domain‐containing protein, uncharacterized protein, DUF641 domain‐containing protein, transposase‐associated domain‐containing protein, and trafficking protein particle complex II‐specific subunit 120 homolog (Table 2). Class VI interacted with GBF‐interacting protein 1 N‐terminal domain‐containing protein, PORR domain‐containing protein, and PAS domain‐containing protein (Table 2). Class VII showed no interactions (Table 2).

Networks involved biological processes including cell‐matrix adhesion, cell migration, receptor signaling, signal transduction, cell shape regulation, actin filament organization, development, cellular process regulation, cell polarity maintenance, GTPase‐mediated signaling, and cytoskeleton organization (Supplementary Table S4). Molecular functions included cell adhesion molecule binding, signaling receptor binding, protein binding, and protein‐containing complex binding (Supplementary Table S4). Actin was a cellular component (Supplementary Table S4).

### 3.7. Analysis of Promoter Cis‐Acting Elements and Functional Prediction

**Figure 8:**
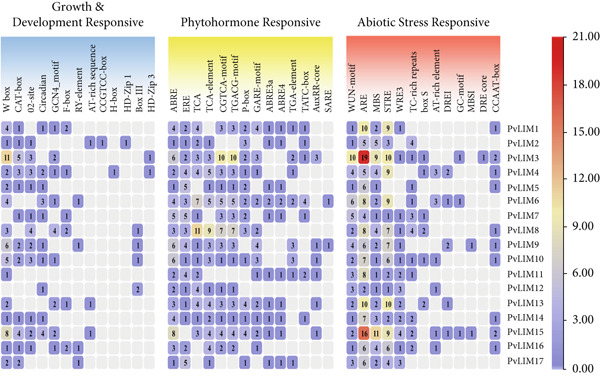
Cis acting elements identified in 17 PvLIM genes.

Twelve development‐related, 13 stress‐related, and 14 hormone‐responsive *cis-*acting elements were identified in PvLIM gene promoters (Figure 8). *Cis-*element patterns showed clear distinctions among groups one to three (based on domain presence, Supplementary Table S7) yet remained consistent within groups (Figure 8).

Development‐related elements involved meristem expression, circadian control, endosperm expression, seed‐specific regulation, LEAFY transcription factor redirection, maximal elicitor‐mediated activation (two copies), meristem‐specific expression activation, root‐specific expression, phytochrome down‐regulation, palisade mesophyll cell differentiation, WRKY binding, and protein binding (Supplementary Table S6).

Hormone‐responsive elements included three abscisic acid‐responsive types—ABRE, ABRE3a, and ABRE4. ABRE occurred in all 17 *PvLIM* genes, while ABRE3a and ABRE4 coexisted in 10 genes (Figure 8, Supplementary Table S6). Additional elements mediated ethylene responsiveness (16 *PvLIM genes*), salicylic acid responsiveness (14 genes), methyl jasmonate (MeJA) responsiveness (14 genes), gibberellin responsiveness (P box in 13 genes, GARE‐motif in 12 genes), and auxin responsiveness (eight genes) (Supplementary Table S5).

Stress‐related *cis-*elements responded to wounding, heat stress, drought, and light stress. Elements for anaerobic induction and anoxic‐specific inducibility suggested roles under low‐oxygen conditions. MYB binding sites regulated dehydration, flavonoid biosynthesis, and stress defense. Among 13 stress‐responsive elements, WUN‐motif, ARE, and MBS occurred in all 17 *PvLIM genes* (Figure 8). STRE appeared in 16 genes (PvLIM5 exception). PvLIM3 contained 19 ARE elements, highlighting unique regulatory complexity (Supplementary Table S5). Phylogenetically clustered *PvLIM genes* shared similar *cis-*acting elements, emphasizing functional conservation within groups (Figures 3, 8).

For the functional prediction of *PvLIM genes*, five unique gene ontology (GO) term annotations were definitively identified and connected with 17 *PvLIM genes* (Table 3). The functional analysis reveals distinct GO term assignments for each gene group. Group I genes are assigned two primary functional categories: cytoskeleton‐related functions and metal ion binding (Supplementary Tables S7, S8). The cytoskeleton‐related functions include GO:0051015 (F‐actin filament binding), actin cross‐linking activity, GO:0051017 (actin filament bundle assembly), actin cable assembly, and actin cable formation, establishing that these genes are crucial for regulating the cytoskeleton (GO:0005856) and maintaining actin structures essential for cellular integrity, shape, and movement. Additionally, Group I genes possess GO:0046872 (metal ion binding), confirming their interactions with metal ions that directly influence protein structure or function. Group II genes are exclusively assigned metal ion binding function (GO:0046872), sharing this single functional category with Group I genes (Table 3, Supplementary Table S7). Group III genes, containing LIM_bind domains, are specifically assigned protein binding functions, including GO:0005515 (protein amino acid binding) and glycoprotein binding, confirming their involvement in protein–protein or protein–glycoprotein complex interactions (Table 3, Supplementary Tables S7, S8).

**Table 3:** Gene ontology annotation.

**GO term**	**Genes associated**		
	**Group I**	**Group II**	**Group III**
GO:0005515	—	—	*PvLIM*3, *PvLIM*9, *PvLIM10*, *PvLIM15*
GO:0005856	*PvLIM*2, *PvLIM*5, *PvLIM*7, *PvLIM11*, *PvLIM12*, *PvLIM14*, *PvLIM16*, *PvLIM17*	—	—
GO:0046872	*PvLIM*2, *PvLIM*5, *PvLIM*7, *PvLIM11*, *PvLIM12*, *PvLIM14*, *PvLIM16*, *PvLIM17*	*PvLIM*1, *PvLIM*2, *PvLIM*4, *PvLIM*5, *PvLIM*6, *PvLIM*7, *PvLIM*8	—
GO:0051015	*PvLIM*2, *PvLIM*5, *PvLIM*7, *PvLIM11*, *PvLIM12*, *PvLIM14*, *PvLIM16*, *PvLIM17*	—	—
GO:0051017	*PvLIM*2, *PvLIM*5, *PvLIM*7, *PvLIM11*, *PvLIM12*, *PvLIM14*, *PvLIM16*, *PvLIM17*	—	—

### 3.8. Expression Data Analysis

**Figure 9:**
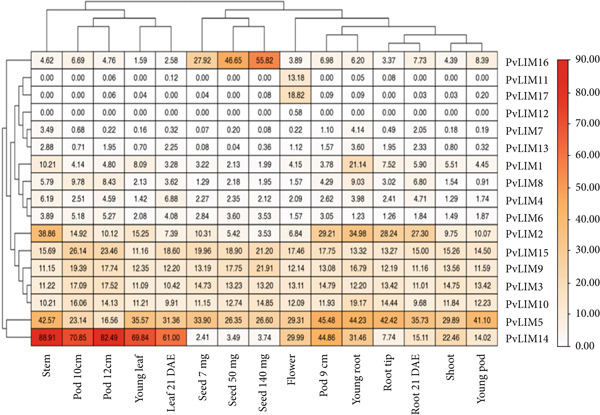
Heat map showing expression level of PvLIM genes in different tissues. Median normalized RPKM values are plotted against respective tissues. Name of the tissues presented are Leaf Young, Leaf 21DAE, Stem, Shoot, Flower, Pod Young, Pod 9 cm, Pod 10 cm, Pod 12 cm, Seed 7 mg, Seed 50 mg, Seed 140 mg, Root Tip, Root Young, Root 21 DAE.

The expression data of selected *PvLIM genes* provide critical insights into their activity across various developmental stages and tissues in the plant. Among the 17 *PvLIM genes*, all except *PvLIM11*, *PvLIM12*, and *PvLIM17* exhibited variable levels of expression across all tissues studied (Figure 9). The comprehensive analysis, which encompassed 15 different tissues, revealed that *PvLIM5* displayed the highest expression in seven tissues, followed by *PvLIM14*, which showed highest expression in six tissues, and *PvLIM16*, which was predominantly expressed in two tissues (Figure 9).

Interestingly, *PvLIM16* exhibited low expression in most tissues except for three seed tissues, where its expression was notably high and positively correlated with increasing seed weight (Figure 9). *PvLIM2* was most prominently expressed in stem tissue, whereas *PvLIM14* demonstrated high expression levels in stem, pod, and leaf tissues, though its expression was relatively low in seed tissues. Genes such as *PvLIM3*, *PvLIM9*, *PvLIM10*, and *PvLIM15* were expressed in all tissues with expression levels ranging from 9.68 to 26.14 (Figure 9). These four genes also belonged to Group III in the classification system developed in this study (Supplementary Table S7).

Group B genes (*PvLIM6*, *PvLIM8*, *PvLIM13*, *PvLIM1*, and *PvLIM4*) showed similar expression patterns across tissues, with *PvLIM1* demonstrating a significantly higher expression in young root tissue. In contrast, *PvLIM11*, *PvLIM12*, and *PvLIM17* exhibited restricted expression patterns, being absent in 10, 14, and 5 tissues, respectively (Figure 9).

Notably, *PvLIM11* was expressed only in root tip, young root, flower, leaf at 21 DAE (days after emergence of the unifoliate leaf), and pod (12 cm stage) tissues. Among these, its expression was particularly high in flowers compared with other tissues, suggesting a potential role in floral biology (Figure 9). This finding underscores the importance of tissue‐specific expression in understanding the functional diversity of the *PvLIM* gene superfamily.

**Figure 10:**
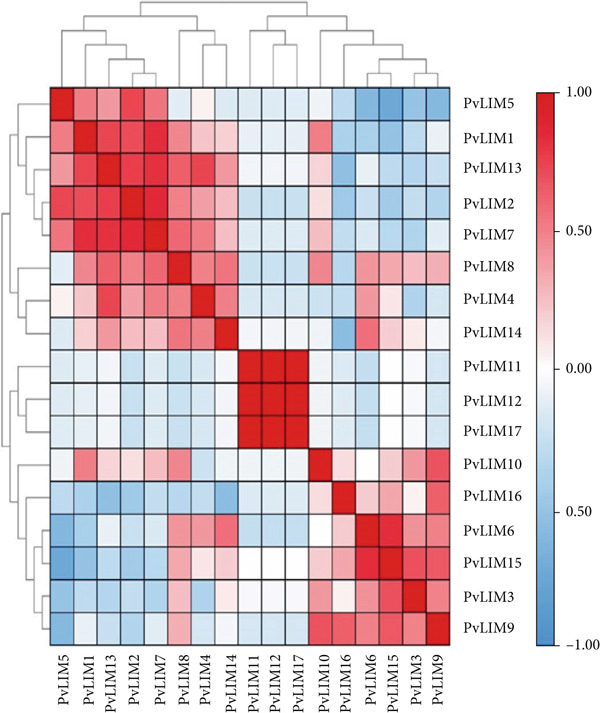
Co‐expression heat map based on Pearson’s correlation coefficient (r). The value is calculated using the RPKM values in leaves of different tissues of *Phaseolus vulgaris* retrieved from the *P. vulgaris* gene expression atlas (no treatment).

The correlation study showed a strong correlation as high as 0.99994 present among the genes (Figure 10). The top five positively co‐expressed gene pairs are *PvLIM11*‐*PvLIM12* (r = 0.99), *PvLIM17*‐*PvLIM12* (r = 0.99), *PvLIM11*‐*PvLIM17* (r = 0.99), *PvLIM*7‐*PvLIM*2 (r = 0.85), and *PvLIM15*‐*PvLIM*6 (r = 0.83) (Supplementary Table S6). While the top five negatively co‐expressed gene pairs are *PvLIM*5‐*PvLIM15* (r = –0.73), *PvLIM*5‐*PvLIM*6 (r = –0.60), *PvLIM*5‐*PvLIM*9 (r = –0.59), *PvLIM16*‐*PvLIM*14 (r = –0.55), and *PvLIM16*‐*PvLIM*13 (r = –0.53) (Supplementary Table S6).

## 4. Discussion

### 4.1. Gene Family Structure and Classification

The LIM domain‐associated gene superfamily in *P. vulgaris* comprises 17 members, representing a moderate‐sized family compared with other species with 21 genes in alfalfa, 15 in tomato, nine in quinoa, six in grapevine, 12 in poplar, six in rice, and six in Arabidopsis, with *G. max* containing 42 *LIM genes* because of its larger genome size (1.1–1.15 Gb) [29, 49–53]. This moderate size ensures optimal functional diversity without excessive genetic redundancy.

This study identifies three *PvLIM* superfamily groups, confirming Groups I and II exhibit classic two‐LIM and one‐LIM architectures while incorporating LIM_bind domain factors (Supplementary Figure S1, Table S7) [3]. Unlike Arabidopsis *LIM genes* with four subgroups (*α*LIM1, *β*LIM1, *γ*LIM2, *δ*LIM2) based on conserved two‐LIM domains, *PvLIM* shows superior structural diversity. Group A divides into A1 and A2 subgroups by interLIM sequence length differences (1–4 amino acids shorter in A2), paralleling established methods [22]. Including LIM_bind domain genes reveals *P. vulgaris* has a more functionally diverse LIM repertoire than traditional Arabidopsis models, providing expanded regulatory capabilities for complex developmental programs.

LIM_bind proteins, including LIM‐domain binding protein 1 (LDB1), function as multi‐adaptor scaffolds that dramatically enhance gene family versatility by assembling dynamic regulatory complexes bridging LIM proteins with diverse transcription factors and coregulators. This scaffolding mechanism enables individual LIM proteins to perform multiple context‐dependent roles spanning gene expression regulation, cytoskeletal organization, and organ development, creating a modular assembly system that drives dynamic regulation of plant development, cell fate, and tissue patterning beyond the capabilities of LIM proteins alone [12, 54]. This architectural innovation allows single genes to control multiple pathways simultaneously.

### 4.2. Validation and Physicochemical Properties

The proposed classification demonstrates exceptional robustness through consistent support from multiple independent analyses including gene structure, phylogenetic analysis with high bootstra*p* values, synteny analysis, protein homology analysis, and functional and expression analyses (Figures 2, 3, 4, 6, 9; Table 3; Supplementary Figure S2). This convergent evidence eliminates classification uncertainty and establishes reliable functional predictions.

The physicochemical properties of LIM proteins show considerable variation in isoelectric pH and GRAVY values due to amino acid sequence differences, with proteins within the same group sharing greater similarities (Table 1, Supplementary Table S7) (L. [55]). The heaviest proteins contain LIM_bind domains, followed by those with DA1_like domains, while the lightest contain double LIM domains, with double LIM and LIM_bind domain proteins being basic in nature versus slightly acidic DA1_like domain proteins (Table 1). These distinct physicochemical signatures directly correlate with functional specialization (Table 3).

### 4.3. Subcellular Localization and Functional Specialization

The subcellular localization patterns of *PvLIM* proteins reveal functionally distinct compartmentalization that directly correlates with their predicted biological roles. Five *PvLIM* proteins localizing to the Golgi apparatus establish a definitive link between these proteins and actin cytoskeleton dynamics, as actin filaments serve as the primary trafficking machinery directing protein transport from the Golgi complex to the central vacuole (Table 1) [56]. Actin‐based movement plays an essential role in transporting vesicles from the Golgi apparatus that carry matrix polysaccharides, while proteins involved in assembling actin patches associate with Golgi‐localized enzymes participating in cell wall biosynthesis [57, 58]. This Golgi localization confirms that *PvLIM genes* directly control cell wall synthesis processes.

The extracellular positioning of certain *PvLIM* proteins demonstrates their integral involvement in cell–cell communication networks and migratory processes [59], establishing these proteins as critical mediators of cellular interactions with the external environment. Most significantly, the exclusive nuclear localization of all Group III proteins (LIM_bind) provides conclusive evidence of their transcriptional regulatory function, as this compartmentalization pattern is necessary and sufficient for their role as gene expression modulators (Table 1). These localization data establish that *PvLIM* protein spatial distribution represents a sophisticated cellular organization system where subcellular positioning directly determines functional specificity.

### 4.4. Chromosomal Distribution and Gene Duplication

Different chromosomal positions of *PvLIM genes* from the same group indicate unlinked independent assortment of the genes (Figure 1). When gene products work together, the variation in phenotypic expression can be explained by combinations created from independent assortment. This creates adaptive advantages by allowing these genes to evolve, be regulated, and respond to selection pressures independently, thereby increasing potential for functional diversity and resilience. This organization reduces risks of harmful interactions from mutations or deletions while enabling metabolically essential growth and developmental roles through molecular‐level execution diversity [60].

Gene pairs like *PvLIM9-PvLIM10*, *PvLIM6-PvLIM8* and others were confidently predicted to undergo duplication events due to their structural similarities, phylogenetic connection, syntenic relationship, and homology models (Supplementary Table S1b). The predicted paralogs show high similarity in gene structure and motif distribution, differing only in intron lengths (Supplementary Table S9, Figure 2). This pattern indicates recent duplication events followed by functional divergence.

Based on *cis-*element patterns in duplicated pairs, the *PvLIM* gene family demonstrates subfunctionalization across five duplicate gene pairs that evolved specialized functions to enhance plant survival and adaptation. *PvLIM15* and *PvLIM3* diverged to handle distinct abiotic stress responses, with *PvLIM15* acquiring specialized ABA‐mediated drought tolerance through dual ABA‐responsive elements while *PvLIM3* evolved gibberellin‐coordinated light stress management. This allows plants to simultaneously withstand water limitation and excessive radiation exposure (Figure 8, Supplementary Table S5).

*PvLIM9* and *PvLIM10* underwent subfunctionalization separating reproductive success from vegetative maintenance, where *PvLIM9* specialized in seed‐specific development and salicylic acid‐mediated pathogen resistance during reproduction while *PvLIM10* retained gibberellin‐driven growth regulation. This enables plants to continue growing under pathogen pressure while protecting reproductive investment. *PvLIM9*’s tissue‐specific role is validated by its higher expression in pod and seed tissues compared with its paralog (Figure 8, Supplementary Table S5).

*PvLIM4* and *PvLIM13* represent developmental versus stress response subfunctionalization, with *PvLIM4* maintaining auxin‐controlled meristem and root development for normal growth while *PvLIM13* evolved integrated ABA‐auxin responsiveness coupled with heat stress and elicitor activation. *PvLIM4* exhibits 214% and 123% higher expression than *PvLIM13* in stem and root tissues, respectively, supporting its tissue‐specific developmental role. Its ~60‐fold elevated expression in seed tissues confirms specialization in reproductive processes (Figure 8, Supplementary Table S5).

*PvLIM8* and *PvLIM6* represent targeted versus comprehensive stress management evolution, where *PvLIM8* developed focused heat and light stress protection through LEAFY transcriptional control while *PvLIM6* became a master stress coordinator integrating multiple hormone pathways and environmental responses. This enables plants to handle both specific threats and complex multi‐stress environments (Figure 8, Supplementary Table S5).

*PvLIM2* and *PvLIM14* suggest temporal and functional partitioning of defense mechanisms, with *PvLIM2* specializing in elicitor‐mediated pathogen defense during active development while *PvLIM14* evolved circadian‐controlled heat stress responses integrated with gibberellin and methyl jasmonate signaling. Both paralogs show elevated expression in stem tissue, with *PvLIM14* exhibiting twice the expression level of *PvLIM2*. In seed tissues, *PvLIM2* surpasses *PvLIM14*, indicating functional shifts between vegetative and reproductive stages (Figure 8, Supplementary Table S5).

### 4.5. Phylogenetic Organization and Subfunctionalization

In the phylogenetic tree, subgroups within each group show similarity in exon–intron distribution within subgroups and dissimilarity among subgroups within groups. Closely positioned genes have similar functions and splicing mechanisms. In Group A, A1 subgroup genes mostly form no interaction network but A2 interacts with Treslin N‐terminal domain‐containing protein, indicating functional specialization post‐divergence.

In Group C, protein interaction analysis reveals that C2 and C1 underwent functional subfunctionalization following divergence. C2 functions as a multi‐regulatory hub through interactions with SWIM, Fip1, DUF641, Transposase‐associated proteins, and TRAPP II components, coordinating transcription, mRNA processing, cell wall signaling, genome stability, and vesicle transport [61–66]. C1 specializes in gene regulatory networks through GBF‐interacting proteins, PORR, and PAS domain factors, focusing on transcriptional control and RNA metabolism in organelles while mediating environmental signaling responses ([67, 68]; M. [69]). This partitioning divides the ancestral gene’s broad regulatory functions between C2’s multi‐process coordination and C1’s specialized organellar–environmental response roles, enhancing regulatory precision and cellular control.

### 4.6. Evolutionary Relationships and Conservation

The synteny mapping establishes evolutionary relationships, with genes from *V. radiata* and *G. max* showing maximum similarity to *PvLIM genes* consistently clustering within identical phylogenetic clades, confirming speciation events and shared ancestry within the Phaseoloid clade that includes *G. max* (soybean), *P. vulgaris* (common bean), *V. unguiculata* (cowpea), and related legumes (Figures 3, 4) [70]. This synteny validates orthologous relationships and functional conservation across legume species.

The phylogenetic analysis reveals a crucial evolutionary pattern: both monocots and dicots are represented across every phylogenetic group, establishing that LIM gene evolution preceded the monocot‐dicot divergence (Figure 3) [51]. This ancient evolutionary origin, combined with orthologous pairs within identical phylogenetic groups, demonstrates that *LIM genes* maintain functional similarity across diverse species, indicating their fundamental importance in plant biology and conservation throughout plant evolution.

Sequence alignment analysis confirms that conserved regions lack complete similarity, indicating multiple mutational events within these regions, with double LIM domains positioned near the N‐terminal in Group I genes and DA1_like domains located near the C‐terminal in Group II proteins (Figure 5). Homology modeling demonstrates that tertiary structures from identical phylogenetic groups exhibit extensive overlapping areas, with *PvLIM* protein models generated primarily from *G. max* and *G. hispada* templates due to maximum homology, further supporting orthologous relationships established through syntenic analysis (Supplementary Figure S2, Table 2; Figure 4).

### 4.7. Protein Interaction Networks and Cellular Functions

*PvLIM* proteins function as integral components in major cellular processes through dynamic gene networks that control cellular structures and biological molecule regulation (Table 3, Figure 7, Supplementary Table S3). Specific *PvLIM* groups demonstrate significant interactions with STIG1 domain proteins, which are developmentally regulated and expressed in stigmatic secretory zones crucial for pollination processes [71]. These proteins facilitate essential cellular functions including intracellular molecule movement and cytoskeletal activity that maintain membrane/wall homeostasis and growth polarity during pollen tube development, where dynamic actin cytoskeleton enables endomembrane trafficking through coordinated crosstalk mechanisms [72].

The protein interaction networks reveal *PvLIM* involvement with WPP domain‐interacting tail‐anchored protein (WIT) for nuclear shape determination and pollen tube guidance, SINE1 for F‐actin association and guard cell nuclear anchorage, and Mu homology domain (MHD) proteins for endocytosis regulation [73–75]. Additionally, *PvLIM* proteins interact with Dolichol‐phosphate mannosyltransferase subunit 1, facilitating N‐glycosylation, glycosyl phosphatidylinositol (GPI) membrane anchoring, and O‐mannosylation processes, alongside Beta‐galactosidase interactions for cell wall modifications (Supplementary Table S3) [76–78]. These comprehensive interactions establish *PvLIM* proteins as central regulators in cell signaling, cell wall metabolism, and essential developmental processes.

Double LIM domain‐containing *PvLIMs* interact with Treslin N‐terminal domain proteins regulating cell division and DNA replication, while contributing to transcriptional and translational regulation through SWIM‐type domain proteins (transcription and DNA repair), Fip1 domain proteins (RNA processing and mRNA stability), and transposase‐associated proteins (transposon activity and genomic rearrangements) (Table 2) ([63]; A. [79, 80]). Their associations with PORR and PAS domain‐containing proteins indicate involvement in stress response and light response, respectively (Table 2) [81]. This broad interaction network positions *PvLIM* proteins as one of the crucial coordinators of cellular homeostasis.

### 4.8. Hormone Responsiveness and Stress Adaptation

All *PvLIM genes* demonstrate strong responsiveness to major plant hormones including abscisic acid (ABA), auxin, gibberellic acid (GA), ethylene, methyl jasmonate (MeJA), and salicylic acid, with ABA‐responsive motifs present in all promoter regions establishing crucial links to ABA‐related plant processes (Figure 8, Supplementary Table S6). Since ABA triggers actin organization changes influencing stomatal closure during stress responses and wound recovery, this universal ABA responsiveness underscores the multifaceted role of *PvLIM genes* in stress adaptation ([82]; Y. [83]). PvLIM7, PvLIM12 and PvLIM16 were found to be upregulated during drought stress [84].

The presence of wound stress response elements across all 17 genes aligns with wound healing’s dependence on actin cytoskeleton‐controlled cellular processes, while drought and anaerobic stress elements emphasize their critical roles under challenging environmental conditions where cytoskeleton and turgor pressure jointly regulate morphogenesis (Figure 8, Supplementary Table S6) ([85]; L. [86]). This comprehensive stress responsiveness makes *PvLIM genes* essential for plant survival under adverse conditions.

### 4.9. Tissue‐Specific Expression and Functional Specialization

Tissue‐specific expression analysis reveals distinct functional specializations: *PvLIM16* drives seed development, *PvLIM11* and *PvLIM17* control flower development exclusively, *PvLIM1* governs young root processes where actin cytoskeleton facilitates environmental responses, and *PvLIM14* regulates vegetative growth [87] (Figure 9). The remaining *PvLIMs* exhibit generalized expression across tissues, contributing to multiple developmental stages with broader physiological roles. *PvLIMs* maintain expression throughout all developmental stages, indicating essential roles in growth and development with differential expression reflecting variable organ‐specific functions (Figure 9). This tissue‐specific expression pattern ensures appropriate gene activity during critical developmental windows.

Expression correlation analysis establishes functional relationships, with positively correlated genes *PvLIM11*, *PvLIM12*, and *PvLIM17* showing near‐zero expression except in flowers, suggesting stress‐related functions supported by *cis-*elements for anaerobic induction, wound response, heat stress, drought‐inducibility (MYB binding sites), and salicylic acid signaling (Figures 8, 9, 10) [88]. Regulatory interactions include *PvLIM15* (containing LIM_bind domain) upregulating *PvLIM6*, while *PvLIM9* and *PvLIM15* LIM_bind domains downregulate *PvLIM5*, highlighting complex feedback mechanisms within the superfamily (Figure 10). These regulatory networks ensure precise control of gene expression under varying conditions.

### 4.10. Biotechnological Implications and Future Applications

These findings establish *PvLIM genes* as key enhancers of both biotic and abiotic stress tolerance. For biotic stress, their wound responsiveness and salicylic acid sensitivity position them as crucial defense components against pathogen penetration through actin cytoskeleton‐mediated barrier formation [89]. For abiotic stress, their ABA responsiveness confirms essential roles in stress adaptation, with actin binding and bundling facilitating cytoskeletal rearrangement responses to drought and environmental challenges [90].

Future overexpression studies and targeted breeding programs can harness *PvLIM* gene potential to develop stress‐tolerant *P. vulgaris* cultivars, establishing them as prime candidates for crop improvement strategies. The comprehensive characterization of this gene family provides the foundation for precision breeding approaches targeting specific stress tolerance traits while maintaining optimal growth and development characteristics.

## 5. Conclusion

This study fundamentally redefines the LIM gene family framework by expanding traditional classification to include LIM_bind domain‐containing factors, revealing that *P. vulgaris* possesses a more functionally versatile regulatory system than previously recognized. The convergence of phylogenetic analysis, gene structure, expression profiling, and protein interaction data validates a robust three‐group classification. *P. vulgaris LIM genes* function as one of the regulators of bridging plant growth and adaptation through sophisticated mechanisms including LIM_bind‐mediated transcriptional scaffolding, strategic subfunctionalization following gene duplication, and comprehensive stress‐hormone integration networks. The identification of tissue‐specific expression patterns (*PvLIM16* in seeds, *PvLIM11/PvLIM17* in flowers, *PvLIM1* in roots), universal ABA responsiveness across all 17 genes, and complex regulatory interactions (*PvLIM15* controlling *PvLIM6* and *PvLIM5* expression) demonstrates how involved these proteins are in coordinating cellular homeostasis, developmental precision, and environmental adaptation simultaneously. Future research should prioritize functional validation of the stress‐responsive *PvLIM14*, reproductive‐specific genes, and LIM_bind regulatory networks through CRISPR‐mediated approaches to unlock their biotechnological potential for developing climate‐resilient cultivars. This comprehensive characterization provides a foundational resource for precision breeding programs targeting enhanced stress tolerance, developmental control, and productivity in common bean, directly supporting sustainable crop improvement under changing environmental conditions.

## Disclosure

All authors have reviewed the final manuscript and agree to be accountable for all aspects of the work presented.

## Conflicts of Interest

The authors declare no conflicts of interest.

## Author Contributions

**Raiyyan Abdur Rahim:** contributed to conceptualization, methodology design, data collection, visualization, formal analysis and preparation of the original draft.

**Maria Chowdhory:** contributed to data collection, visualization and formal analysis.

**Mahmuda Akhter Hasi:** contributed to data collection, visualization and formal analysis.

**Fabiha Haque:** contributed to data collection, visualization and formal analysis.

**Arif Hasan Khan Robin:** responsible for conceptualization, methodology design, supervision of the project and editing the manuscript.

## Funding

No funding was received for this manuscript.

## Supporting Information

Additional supporting information can be found online in the Supporting Information section.

## Supporting information


**Supporting Information 1** Supplementary Table S1: Gene duplication. Supplementary Table S2: Protein homology modeling. Supplementary Table S3: Protein network information. Supplementary Table S4: Functions of protein network. Supplementary Table S5: Functions of *cis-*elements. Supplementary Table S6: Coexpression table. Supplementary Table S7: Gene domain information. Supplementary Table S8: Annotated function. Supplementary Table S9: Motif information. Supplementary Figure S1: Basic gene model of three identified groups of *PvLIM* genes. Supplementary Figure S2: *PvLIM* genes protein homology models super positioned according to groups created from similar proteins.

**Supporting Information 2** Supplementary Dataset 1.

**Supporting Information 3** Supplementary Dataset 2.

## Data Availability

The data that support the findings of this study are available from the corresponding author upon reasonable request.
